# Glutamine Modulates Expression and Function of Glucose 6-Phosphate Dehydrogenase via NRF2 in Colon Cancer Cells

**DOI:** 10.3390/antiox10091349

**Published:** 2021-08-25

**Authors:** Ibrahim H. Polat, Míriam Tarrado-Castellarnau, Adrian Benito, Claudia Hernandez-Carro, Josep Centelles, Silvia Marin, Marta Cascante

**Affiliations:** 1 Department of Biochemistry and Molecular Biomedicine, Faculty of Biology, Universitat de Barcelona, Av Diagonal 643, 08028 Barcelona, Spain; polat@med.uni-frankfurt.de (I.H.P.); mtarrado@ub.edu (M.T.-C.); a.benito-mauricio@imperial.ac.uk (A.B.); claudiahernandez@ub.edu (C.H.-C.); josepcentelles@ub.edu (J.C.); 2 Institute of Biomedicine, Universitat de Barcelona (IBUB), 08028 Barcelona, Spain; 3 Equipe Environnement et Prédiction de la Santé des Populations, Laboratoire TIMC (UMR 5525), CHU de Grenoble, Université Grenoble Alpes, CEDEX, 38700 La Tronche, France; 4 CIBER of Hepatic and Digestive Diseases (CIBEREHD), Institute of Health Carlos III (ISCIII), 28029 Madrid, Spain

**Keywords:** cancer cell metabolism, glucose-6-phosphate dehydrogenase, pentose phosphate pathway, colon cancer, oxidative stress

## Abstract

Nucleotide pools need to be constantly replenished in cancer cells to support cell proliferation. The synthesis of nucleotides requires glutamine and 5-phosphoribosyl-1-pyrophosphate produced from ribose-5-phosphate via the oxidative branch of the pentose phosphate pathway (ox-PPP). Both PPP and glutamine also play a key role in maintaining the redox status of cancer cells. Enhanced glutamine metabolism and increased glucose 6-phosphate dehydrogenase (G6PD) expression have been related to a malignant phenotype in tumors. However, the association between G6PD overexpression and glutamine consumption in cancer cell proliferation is still incompletely understood. In this study, we demonstrated that both inhibition of G6PD and glutamine deprivation decrease the proliferation of colon cancer cells and induce cell cycle arrest and apoptosis. Moreover, we unveiled that glutamine deprivation induce an increase of G6PD expression that is mediated through the activation of the nuclear factor (erythroid-derived 2)-like 2 (NRF2). This crosstalk between G6PD and glutamine points out the potential of combined therapies targeting oxidative PPP enzymes and glutamine catabolism to combat colon cancer.

## 1. Introduction

In many tumors, metabolism is strictly reprogrammed to generate energy and biomolecules required for uncontrolled growth that defines cancer [1,2]. There are different strategies to counteract metabolic changes associated with cancer cell proliferation [3]. Even though glycolysis forms the backbone of central carbon metabolism, proliferating cells also highly rely on the pentose phosphate pathway (PPP) in order to synthesize nucleotides for DNA replication and RNA synthesis. PPP provides an alternative route to glycolysis for the metabolism of glucose, and the percentage of glucose metabolized through PPP is known to vary from 5 to 30% depending on the tissue type [4]. Both DNA and RNA are polymers of nucleotides, each of which requires a pentose sugar (deoxyribose for DNA and ribose for RNA) obtained via the PPP, giving this pathway an essential role in nucleotide synthesis. Furthermore, PPP also produces the reducing equivalents, NADPH, which not only are involved in the regulation of reactive oxygen species (ROS) through the maintenance of reduced glutathione (GSH) pool, but also serve as cofactors in the biosynthesis of several essential macromolecules, such as lipids and amino acids [5,6,7]. Besides serving as a crucial pathway for the biosynthesis and the maintenance of redox status, PPP also plays important roles in various aspects related to cancer cells viability, including proliferation, apoptosis, invasiveness, drug resistance, and metastasis [4,8,9,10]. Cancer cells are known to be significantly dependent on PPP to maintain their highly proliferative state [6,11,12]. The relation between elevated PPP and tumor proliferation has been widely studied in several cancer models since PPP mediates cancer cells to meet their anabolic needs together with overcoming oxidative stress [8,9,10,13,14,15].

PPP consists of two different branches that converge in the production of ribose-5-phosphate, which is essential for the synthesis of nucleotides. The oxidative branch of PPP (ox-PPP) is a non-reversible metabolic pathway that starts with the transformation of glucose-6-phosphate (G6P) into 6-phosphoglucono-δ-lactone. This reaction is catalyzed by the glucose-6-phosphate dehydrogenase (G6PD) enzyme. G6PD catalyzes the rate-limiting step in the ox-PPP that generates the first molecule of NADPH; so, its expression and activity are tightly regulated [4]. G6PD usually works at 1–2% of its maximal potential in healthy cells, as NADPH concentration in the quiescent condition is high. However, similar to the tissues with an active metabolism such as liver, adipose, or mammary glands, tumor cells, including colon cancer cells, are reported to have high levels of G6PD due to their higher consumption of NADPH compared to quiescent cells [16,17,18].

PPP is regulated by oncogenes and tumor suppressor genes. *KRAS* (which is a subfamily isoform of RAS oncogene) and *PI3K*, two of the most frequently mutated oncogenes, increase the activity of G6PD [19,20]; whereas, tumor suppressor gene *TP53* downregulates G6PD activity by decreasing its stability upon directly binding to it [21]. Moreover, transcription factors in response to cellular stress, such as nuclear factor (erythroid-derived 2)-like 2 (NRF2), have also been reported to regulate G6PD activity [22,23]. NRF2 plays a key role in tumorigenesis since it is usually upregulated in several cancer types triggering in turn upregulation of its target genes such as G6PD, malic enzyme 1 (ME1), and isocitrate dehydrogenase 1 (IDH1) [22,24,25].

Glutamine is a non-essential amino acid with several cellular functions, including the donation of nitrogen for nucleotide and protein synthesis, energy production, and lipid and non-essential amino acids synthesis [26,27,28]. In particular, the synthesis of nucleotides requires glutamine and 5-phosphoribosyl-1-pyrophosphate produced from ribose-5-phosphate via the oxidative branch of the PPP [29]. Also, glutamine is converted to glutamate by glutaminase (GLS) either to be used as a precursor of GSH and non-essential amino acids, such as aspartate, proline, alanine, and arginine, or to be converted to alpha ketoglutarate (αKG) by glutamate dehydrogenase (GDH) or transaminases to foster the tricarboxylic acid (TCA) cycle. αKG is further oxidized to malate and might leave the TCA cycle through conversion to pyruvate by the malic enzyme (ME), which also produces NADPH [28]. When glutamine is oxidized to pyruvate, the derived NADPH allows tumor cells to reduce the ROS associated with mitochondrial respiration and rapid cell proliferation. In addition, as an alternative to glycolysis, glutamine oxidation provides tumor cells with the precursors for major anaplerotic processes such as TCA cycle intermediates to fulfill their bioenergetic and metabolic needs. Similar to PPP, glutamine metabolism is also involved in redox detoxification and nucleotide synthesis [26,27,28], implying a possible crosstalk between both pathways.

In the previous studies performed within our team using breast cancer cell lines, we demonstrated that the inhibition of glucose 6-phosphate dehydrogenase (G6PD), the first enzyme of ox-PPP, leads to a decrease in cell proliferation and alterations in the central carbon metabolism [10]. Similarly, we found out that 6-phosphogluconate dehydrogenase (6PGD), the third enzyme of ox-PPP, also has significant importance in the proliferation of breast cancer cells. In particular, we observed a significant link between PPP and glutamine metabolism in breast cancer cells, since the inhibition of the 6PGD enzyme led to enhanced glutaminolysis and increased activities of some enzymes involved in glutamine metabolism such as the malic enzyme (ME) and isocitrate dehydrogenase (IDH) [9].

In this study, we investigated the effect of G6PD inhibition and the link between glutamine metabolism and PPP in colon cancer cells, taking into account the high reliance of these types of cancer cells on PPP [6]. We demonstrated the fundamental role of the key enzyme of the oxidative branch of PPP and glutamine on proliferation and other phenotypic traits of colon cancer cells. Finally, we also validated the cross-regulation between PPP and glutamine metabolism in colon cancer cells that we previously observed [9,10] in breast cancer cells.

## 2. Materials and Methods

### 2.1. Cell Culture and Cell Proliferation

Colon cancer cell lines HT29, HCT116, SW620, SW480, and Caco-2 were purchased from the American Type Culture Collection (ATCC, Manassas, VA, USA). NCM460 human epithelial cells derived from healthy colon mucosa [30] were a kind gift from Mary Pat Moyer (INCELL, San Antonio, TX, USA). All cell lines were regularly tested for mycoplasma contamination. HT29 and Caco-2 cells were cultured in DMEM (Gibco, Thermo Fisher Scientific, Waltham, MA, USA) containing 10% fetal bovine serum (Gibco, Thermo Fisher Scientific, Waltham, MA, USA), 10 mM d-glucose (Sigma-Aldrich, MA, USA), and 2 mM l-glutamine (Gibco, Thermo Fisher Scientific, Waltham, MA, USA). HCT116 cell line was cultured in the mixture of DMEM (Gibco, Thermo Fisher Scientific, Waltham, MA, USA) and HAM-F12 (Biowest, Nuaillé, France) (1:1 *v*/*v*) containing 10% fetal bovine serum (Gibco, Thermo Fisher Scientific, Waltham, MA, USA), 12.5 mM d-glucose (Sigma-Aldrich, MA, USA), 2 mM l-glutamine (Gibco). SW620 and SW480 cells were grown in DMEM with 12.5 mM d-glucose, 4 mM glutamine, 5% fetal bovine serum (Gibco). NCM460 cells were grown in M3Base medium (INCELL) with 5 mM d-glucose (Sigma-Aldrich) and 2 mM l-glutamine (Gibco). All media were supplemented with 1% antibiotic (penicillin 100 Units/mL-streptomycin 100 µg/mL, Gibco, Thermo Fisher Scientific, Waltham, MA, USA). The cells were maintained at 37 °C with 5% CO_2_ and saturated humidity. The growth medium was replaced every 2–3 days and the cells were passed before they reached 80% confluence.

The proliferation kinetics and viability of the transfected cells were measured using flow cytometry combining direct cell counting and propidium iodide (PI) staining. Shortly, cells were washed with phosphate buffered saline (PBS), trypsinized, and resuspended in their corresponding medium. Just before measurement flow-count fluorospheres and PI were added. The analysis was performed using a Beckman Coulter^®^ Epics^®^ XLTM Flow Cytometer (Beckman Coulter, Indianapolis, IN, United States) adjusted to 1 × 10^4^ fluorospheres cut-off. The total cell number was registered, allowing discrimination between dead and alive cells.

### 2.2. Chemicals

Telaglenastat (CB-839) (CAS No. 1439399-58-2), R162 (CAS No. 64302-87-0), and GPNA Hydrochloride (CAS No. 67953-08-6) were purchased from MedChemExpress (Monmouth Junction, NJ, USA) and BPTES (CAS No. 314045-39-1) from Sigma-Aldrich (Milwaukee, WI, USA).

### 2.3. siRNA Transfection

For the transfection of HT29 and HCT116 cell lines, the cells were seeded at a density of 5 × 10^4^ cells per well in a 6-well plate with an antibiotic-free growth medium. After 24 h, they were transfected in triplicates with 50 nM for HT29 cells and 10 nM for HCT116 cells of either siNEG pool or siRNA pool against G6PD using Lipofectamine RNAiMAX (Invitrogen, Darmstadt, Germany) according to the manufacturer’s protocol. The quantity of siRNA used was optimized for each cell line. The medium was replaced after 6 h with a complete medium containing antibiotics as well. The siRNA pool targeted against G6PD was purchased from Dharmacon (Lafayette, CO, USA) and is listed as follows: siG6PD, ON-TARGETPlus SMARTpool L-008181-02-0010 with the sequences: ACAGAUACAAGAACGUGAA; CCGUGUACACCAACAUGAU; CAGAUAGGCUGGAACCGCA; AUUCACGAGUCCUGCAUGA. Control siRNA pool (siNEG) was also purchased from Dharmacon (Lafayette, CO, USA): siNEG ON-TARGET Plus Non-Targeting siRNA D-001810-10-20 (Sequence not provided by the manufacturer).

### 2.4. RNA Isolation and Gene Expression Analysis

RNA isolation from the transfected cells from fresh or frozen plates was done using Trizol^®^ reagent (Sigma, Marlborough, MA, USA) according to the manufacturer’s protocol. The conversion of RNA into cDNA was done using 1 μg of RNA, random primers (Roche, Basel, Switzerland), and M-MLV reverse transcriptase enzyme (Invitrogen, Darmstadt, Germany) according to the manufacturer’s protocol. Gene expression analysis was performed by real time polymerase chain reaction (RT-PCR) (Applied Biosystems^®^ 7500 Real Time PCR, Applied Biosystems, Darmstadt, Germany) in standard conditions provided by the manufacturer employing Taqman^®^ (Applied Biosystems, Thermo Fisher Scientific, Darmstadt, Germany) gene-specific probes for G6PD, NRF2, HMOX1, and NQO1. The expression levels were quantified using the ΔΔCt method using peptidylprolyl isomerase A (PPIA) as a reference gene.

### 2.5. Glucose 6 Phosphate Dehydrogenase Enzyme Activity Assay

Fresh cell culture plates were rinsed with PBS and lysed with lysis buffer (20 mM tris-HCl, pH 7.5, 1 mM dithiothreitol, 1 mM EDTA, 0.02% (*v*/*v*) triton X-100, 0.02% (*v*/*v*) sodium deoxycholate) supplemented with protease and phosphatase inhibitor cocktails (Thermo Fisher Scientific, Darmstadt, Germany). The cells were scraped, and the cell lysate was disrupted by sonication using a titanium probe (Vibracell, Sonics & Materials Inc., Newtown, US; Tune 50, Output 20, 3 cycles of 5 s each) and centrifuged at 12,000× *g* at 4 °C for 20 min. The supernatant was separated and immediately used to determine specific enzyme activities using the COBAS Mira Plus analyzer (Horiba ABX, Kyoto, Japan). Enzymatic activities were determined by monitoring the increase or decrease of absorbance due to NAD(P)H at 340 nm wavelength. The enzyme activity for each sample was then normalized to the total protein content of the samples measured by BCA assay at 550 nm (Pierce, Thermo Fisher Scientific, Waltham, MA, USA). Specific activities of G6PD were measured by adding samples to a cuvette containing 0.5 mM NADP^+^ in 50 mM tris-HCl, pH 7.6, at 37 °C. The reaction was initiated by the addition of glucose-6-phosphate (G6P) up to a final concentration of 2 mM.

### 2.6. Cell Cycle Distribution Analysis

For cell cycle analysis, the transfected cells were harvested after 96 h, resuspended in 200 μL of 1× TBS buffer, fixed and stained with 200 μL of vindelov-PI solution, and incubated at room temperature for 30 min in the dark. The analysis was performed using a Beckman Coulter^®^ Epics^®^ XLTM Flow Cytometer (Beckman Coulter, Indianapolis, IN, United States) with a cut-off at 1 × 10^4^ cells. Cell cycle distribution analysis was done using FlowJo^®^ software (Version 7.1. Becton, Dickinson & Company, Ashland, OR, USA), through which the percentage of cells in G1, S, and G2 phases was obtained.

### 2.7. Western Blot

Protein extracts were obtained from either fresh or frozen plates 96 h after transfection using the protocol described for the enzyme activity assays. The protein level in each sample was quantified using the BCA assay according to the manufacturer’s protocol. Western blot analysis was carried out using 30 μg of protein, and after electrophoretic separation, proteins were transferred onto a PVDF membrane (Bio-Rad Laboratories, Feldkirchen, Germany). The membranes were then blocked with 0.5% of non-fat dry milk in 0.1% PBS-Tween, and then incubated with G6PD (ab993; Abcam, Cambridge, UK), NRF2 (sc-365949; Santa Cruz Biotechnology, Santa Cruz, CA, USA) or β-actin (#69100; MP Biomedicals, Santa Ana, CA, USA) followed by exposure to corresponding anti-mouse (GR304350-1, Abcam, Cambridge, UK) or anti-rabbit (GR297013-4, Abcam, Cambridge, UK) horseradish peroxidase-conjugated secondary antibody. Visualization was carried out on Fujifilm X-ray (Fuji Medical X-ray Film, Dusseldorf, Germany) using chemiluminescence detection.

### 2.8. Intracellular ROS Level Measurement

Total intracellular ROS levels were determined using flow cytometry and an H_2_DCFA probe (Sigma, Marlboroughcity, MA, USA). The cells were incubated with 5 μM H_2_DCFA in PBS for 30 min. Afterward, PBS was replaced with a complete growth medium, and the cells were incubated for 15 min at 37 °C and 5% CO_2_. Next, cells were trypsinized and resuspended in a solution containing 50 μM H_2_DCFA and 20 μg/ml propidium iodide. Internalized probes reacted with ROS and emitted fluorescence when excited at 492 nm. Emitted fluorescence was recorded by a flow cytometer (Beckman Coulter^®^ Epics^®^ XLTM, Beckman Coulter, Indianapolis, IN, United States) at 520 nm wavelength with a cut-off range of 1 × 10^4^ cells. For the ROS analysis, only PI-negative cells were taken into consideration.

### 2.9. Statistical Analysis

For statistical analysis, parametric unpaired two-tailed independent samples Student’s *t*-test was used. In all figures, bars represent the mean of triplicates ± standard deviation (SD). Statistical significance was assumed if a null hypothesis could be rejected when at least *p* < 0.05 for a confidence interval of >95%. One asterisk (*) denotes *p*-value < 0.05, two asterisks (**) denote *p*-value < 0.01 and three asterisks (***) denote *p*-value < 0.001.

## 3. Results

### 3.1. G6PD Inhibition Alters the Proliferation of HT29 and HCT116 Cells

**Figure 1:**
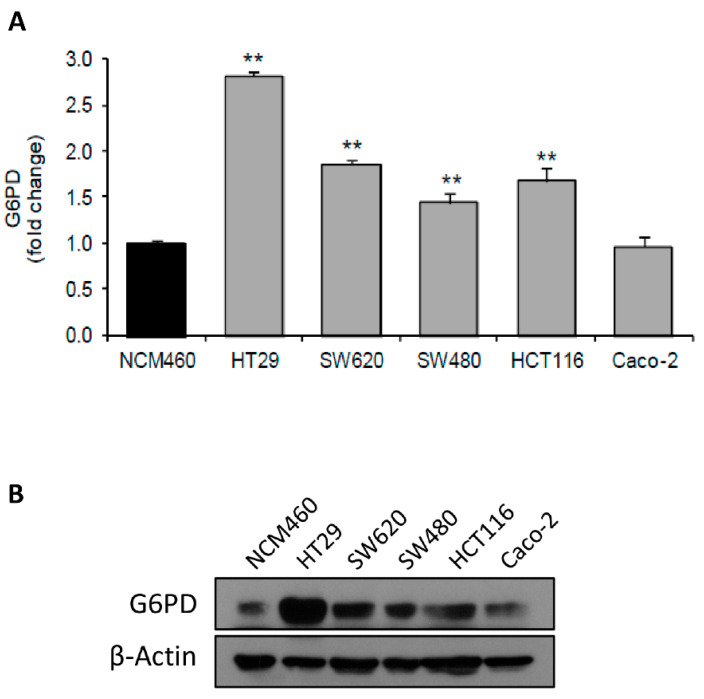
Colon cancer cells have greater glucose-6-phosphate dehydrogenase (G6PD) specific activity than epithelial cells derived from healthy colon mucosa. (**A**) G6PD specific activity and (**B**) protein levels in a panel of colon cancer cell lines. Enzyme activity results are expressed as a percentage of G6PD activity with respect to non-tumor colon cell line NCM460. Two asterisks (**) denote *p*-value < 0.01.

We first measured the specific enzyme activity of G6PD in a panel of colon cancer cells and observed that it was highly upregulated in colon cancer cells compared to non-tumor NCM460 colon cells (Figure 1A). In addition, we assessed G6PD protein levels in the same cell lines by western blot (Figure 1B and Supplementary Figure S1A). Considering that microsatellite instability (MSI) is a marker of chemoresistance associated with improved survival compared with microsatellite-stable (MSS) colon cancers, we wanted to study the effects of G6PD depletion in both genetic conditions as tumors respond differently to chemotherapy depending on this status [31,32]. The results showed that HT29 (MSS), HCT116 (MSI), SW620 (MSS), and SW480 (MSS) displayed the highest G6PD specific activity among the tested cell lines, as well as the highest G6PD protein levels, especially HT29. Accordingly, we selected HT29 and HCT116 cell lines to further characterize the function of G6PD in colon cancer.

**Figure 2:**
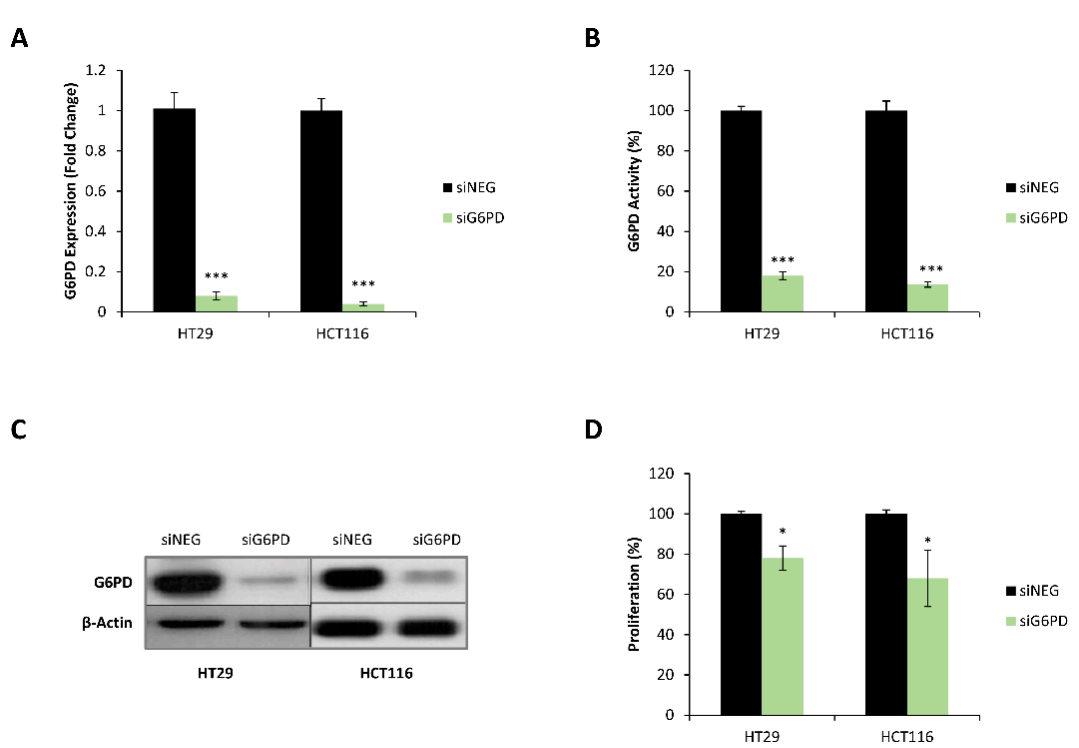
G6PD knockdown caused the inhibition of G6PD activity and reduced the proliferation of colon cancer cells. (**A**) G6PD mRNA expression 72 h after transfection with non-targeting siRNA pool (siNEG) or siRNA pool against G6PD. Fold change was calculated with respect to cells transfected with the siNEG pool. (**B**) Total G6PD enzyme activity normalized to intracellular protein content measured 96 h after transfection using either non-targeting siRNA pool or siRNA pool against G6PD. Fold change was quantified relative to cells transfected with the siNEG pool. (**C**) G6PD protein levels 96 h post-transfection using either non-targeting siRNA pool or siRNA pool against G6PD. (**D**) Effect of G6PD knockdown on cell proliferation measured by flow cytometry 120 h after transfection for G6PD-inhibited cells and cells transfected with siNEG pool. Fold change was quantified relative to cells transfected with the siNEG pool. One asterisk (*) denotes *p*-value < 0.05, three asterisks (***) denote *p*-value < 0.001.

To test the reliance of colon cancer cells on the oxidative phase of PPP for proliferation and other cellular functions, we inhibited *G6PD* using a pool of small interference RNA (siRNA) containing four different sequences targeting different exonic regions of the *G6PD* gene (siG6PD). In order to obtain a relative comparison, we used a negative control (siNEG) which also contains a pool of four different siRNA sequences that did not target any specific region of the genome. The analysis of *G6PD* gene expression 72 h after transfection with siG6PD confirmed a successful inhibition at the mRNA level in HT29 and HCT116 cells, with a decrease of more than 90% compared to control cells transfected with non-targeting siRNA pool (Figure 2A). Moreover, the inhibition of G6PD at the protein level was assessed 96 h after transfection by measuring the specific enzyme activity and further confirmed through western blot. We found that in both HT29 and HCT116 cell lines, the siG6PD pool decreased G6PD enzyme activity by over 80%, and western blot analysis demonstrated a visible decrease in the protein levels of this enzyme (Figure 2B,C and Supplementary Figure S1B).

Several studies have shown that PPP has an essential role in cell growth and proliferation [9,10,33,34,35,36]. Considering this, we examined the role of the G6PD enzyme in the proliferation of colon cancer cell models. Significantly, G6PD knockdown caused a reduction of approximately 25% in the proliferation of HT29 and HCT116 cells compared to control cells 120 h after transfection with siG6PD (Figure 2D). This result indicates that HT29 and HCT116 colon cancer cells with reduced G6PD activity have a decreased proliferation rate compared to those with fully functional PPP.

### 3.2. Glutamine Deprivation Reduces Cell Proliferation and Leads to Cell Cycle Arrest and Apoptosis

**Figure 3:**
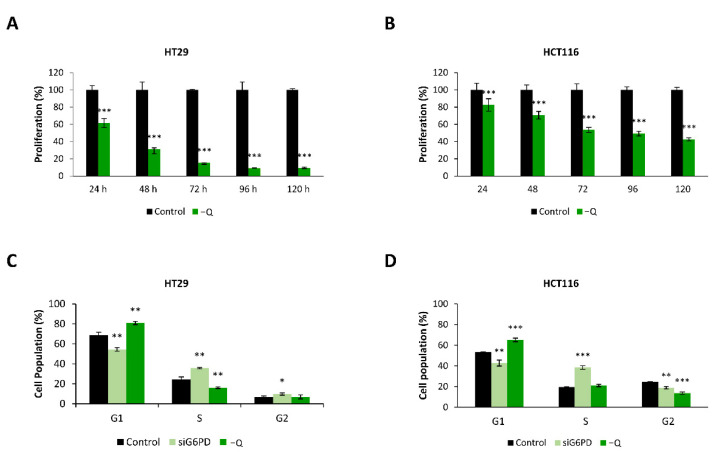
Glutamine deprivation decreased the proliferation of colon cancer cells and induced cell cycle arrest. (**A**,**B**) Proliferation rate of (**A**) HT29 cells and (**B**) HCT116 cells in control media and glutamine-free media (−Q) at 24, 48, 72, 96, and 120 h. (**C**,**D**) Cell cycle distribution analysis of (**C**) HT29 and (**D**) HCT116 cells 96 h after transfection against G6PD or glutamine deprivation. The percentage of cells in the different cell cycle phases was calculated using FlowJo^®^ software. One asterisk (*) denotes *p*-value < 0.05, two asterisks (**) denote *p*-value < 0.01 and three asterisks (***) denote *p*-value < 0.001.

NADPH used to cope with cellular stress is produced through several metabolic pathways, including PPP [4,18] and glutamine metabolism [37]. As the inhibition of oxidative PPP decreased cell proliferation, we wanted to explore the effect of glutamine deprivation on colon cancer cells. To this end, the cells were cultured in glutamine-free media and proliferation was monitored for five days. As shown in Figure 3A,B, both HT29 and HCT116 colon cancer cells cultured in glutamine-free media exhibited a reduced proliferation rate compared to cells cultured in a complete medium.

Next, we wanted to better explore the mechanism through which G6PD inhibition and glutamine deprivation reduced colon cancer cell proliferation. It is known that PPP is essential for the biosynthesis of nucleotides required for DNA synthesis, thus playing an important role in cell cycle progression. G6PD has been described to be regulated through the cell cycle, showing the highest activity at G1 and S phases [8,38]. Similarly, glutamine metabolism has also been linked to the cell cycle machinery through redox detoxification, nucleotide biosynthesis, and other metabolic activities [39]. Therefore, we speculated that the G6PD enzyme and glutamine availability might play an important role in the cell cycle progression. To test this hypothesis, we analyzed the population of both HT29 and HCT116 cells in each cell cycle phase upon *G6PD* knockdown or glutamine deprivation. To do this, 96 h after transfection with siG6PD/siNEG pools or glutamine withdrawal, the cells were stained with vindelov-PI solution after fixation, and DNA content was quantified by flow cytometry. The analysis of the cell cycle distribution 96 h after G6PD knockdown indicated a significant arrest in S phase and a subsequent decrease in G1 phase in both cell lines. On the contrary, glutamine withdrawal led both cell lines to an arrest in the G1 phase (Figure 3C,D and Supplementary Figure S2).

### 3.3. Glutamine Availability Modulates G6PD through NRF2 Activation

**Figure 4:**
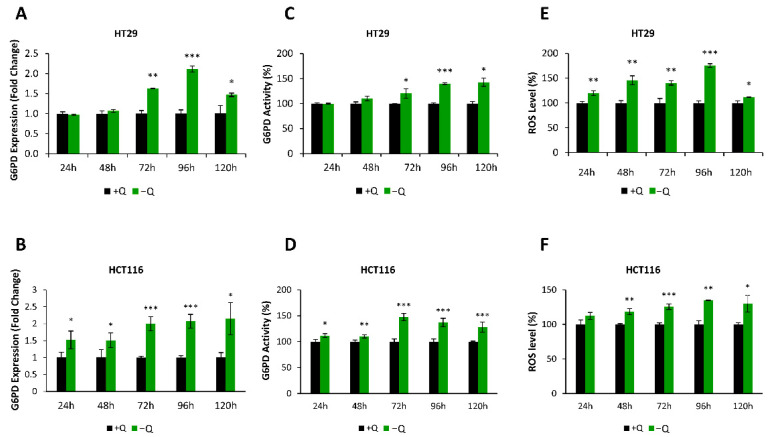
G6PD is modulated by glutamine availability. (**A**,**B**) *G6PD* mRNA expression at different time points after glutamine deprivation in (**A**) HT29 and (**B**) HCT116 cells (−Q). Fold change was calculated with respect to cells cultured in a medium containing glutamine (+Q). (**C**,**D**) Total G6PD enzyme activity normalized to intracellular protein content measured at different time points after glutamine deprivation in (**C**) HT29 and (**D**) HCT116 cells. Fold change was calculated relative to cells cultured in a medium containing glutamine. (**E**,**F**) Relative ROS levels measured at different time points by flow cytometry using H_2_DCFA probes. ROS levels of (**E**) HT29 and (**F**) HCT116 cells cultured without glutamine (−Q) are compared as fold change with respect to cells cultured with glutamine (+Q). One asterisk (*) denotes *p*-value < 0.05, two asterisks (**) denote *p*-value < 0.01 and three asterisks (***) denote *p*-value < 0.001.

Since in our previous studies, we observed increased glutamine consumption in breast cancer cells with reduced G6PD activity [10], we aimed to investigate whether *G6PD* expression is modulated by glutamine deprivation. Therefore, we cultured HT29 and HCT116 cells in a glutamine-free medium for several time points and measured the expression levels of the *G6PD* gene. Interestingly, we observed a significant increase in *G6PD* expression in the absence of glutamine in HT29 and HCT116 cells that was paralleled by changes in enzyme activity (Figure 4A–D). These results show evidence of a metabolic relation between G6PD and glutamine metabolism in colon cancer cell models. 

Then, we wanted to explore the mechanism underlying the increase of *G6PD* expression after glutamine deprivation in colon cancer cells. Nuclear factor (erythroid-derived 2)-like 2 (NRF2) protein is a transcription factor that regulates the expression of genes encoding antioxidant proteins that protect cells against oxidative damage [40]. Also, it has been reported that cells with an activated RAS pathway, such as HT29 cells, have constitutively elevated expression levels of *NRF2* [24,41]. Moreover, it is known that G6PD is regulated by the NRF2 transcription factor [22,23]. In fact, NRF2 is further regulated by Kelch-like ECH-associated protein 1 (KEAP1) in a way that under normal circumstances, NRF2 is constantly ubiquitinated by KEAP1 for its degradation; however, under oxidative stress conditions, KEAP1 is inactivated, and NRF2 migrates to the nucleus to activate an antioxidant response program involving several PPP genes [23,42]. Given that we showed that glutamine deprivation enhances *G6PD* expression in HT29 cells, we hypothesized that glutamine deprivation might induce an increase of intracellular ROS levels that would promote a transcriptional program modulated by NRF2.

To test this hypothesis, we first wanted to confirm whether glutamine deprivation increases ROS levels in colon cancer cells. We measured intracellular ROS production using H_2_DCFA probes in cells deprived of glutamine (Figure 4E,F). Starting from as early as 24 h of glutamine deprivation, we observed a significant and gradual increase in ROS levels, indicating that glutamine is involved in the maintenance of the redox status of this cell line. In redox detoxification, as mentioned earlier, glutamine is implicated not only in NADPH production but also in glutathione (GSH) production [37]. Therefore, the elevated ROS levels observed in HT29 cells with glutamine deprivation are expected and reinforce our hypothesis.

**Figure 5:**
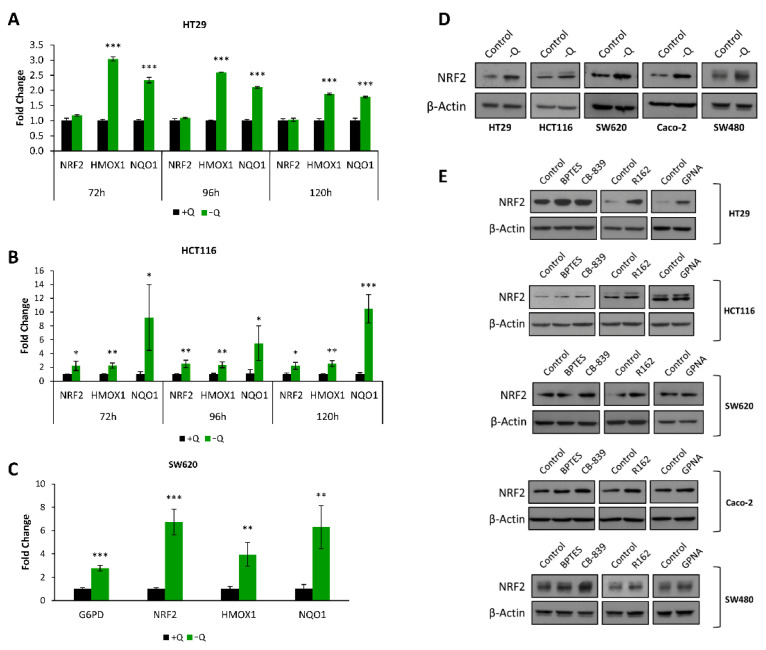
NRF2 and its targeting genes are modulated by glutamine availability. (**A**,**B**) *NRF2*, *HMOX1* and *NQO1* mRNA expression levels 72, 96 and 120 h after depriving (**A**) HT29 and (**B**) HCT116 cells of glutamine. (**C**) *G6PD*, *NRF2*, *HMOX1*, and *NQO1* mRNA expression levels 96 h after depriving SW620 cells of glutamine. Fold change was calculated with respect to cells cultured with a medium containing glutamine. (**D**) NRF2 protein levels 24 h after withdrawal of glutamine from the culture medium. (**E**) NRF2 protein levels after 24 h-treatment with BPTES (10 µM), CB-839 (5 µM), R162 (20 µM), and GPNA (100 µM). One asterisk (*) denotes *p*-value < 0.05, two asterisks (**) denote *p*-value < 0.01 and three asterisks (***) denote *p*-value < 0.001.

Next, to explore the NRF2 activation under glutamine deprivation, we measured the expression level of the *NRF2* gene (codified as NFE2L2) and some other validated NRF2 target genes; such as *NQO1 (NAD(P)H Quinone Dehydrogenase 1)* [22] and *HMOX1* (*Heme oxygenase (decycling) 1*) [24], in both HT29 and HCT116 cells cultured with or without glutamine by using quantitative real-time PCR. We found out that, in cells cultured without glutamine, not only *G6PD* (see Figure 4A,B and Figure 5C) but also both NRF2 target genes (*HMOX1* and *NQO1*) were upregulated (Figure 5A–C). The expression levels of *NRF2* did not significantly change in HT29 cells while they were also upregulated in both HCT116 and SW620 cells (Figure 5A–C). Taking into account that KEAP1 regulates NRF2 by ubiquitination and protein degradation, we speculated that NRF2 might be upregulated at the protein level in the absence of glutamine. To test this hypothesis, a panel of colon cancer cells was grown in the absence of glutamine for 24 h, and NRF2 protein levels were investigated by western blot. Figure 5D and Supplementary Figure S3 show that the absence of glutamine upregulated NRF2 protein levels in HT29, HCT116, SW620, Caco-2, and SW480 cell lines. These results demonstrate the activation of a genetic response mediated by NRF2 in the absence of glutamine in colon cancer cells. To determine whether the specific inhibition of glutamine catabolism can trigger the upregulation of NRF2 as observed in the absence of glutamine, we treated the cells for 24 h with two specific inhibitors of glutaminase, BPTES and CB-839; an inhibitor of glutamate dehydrogenase, R162; and an inhibitor of the glutamine transporter ASCT2 (SLC1A5), GPNA (Figure 5E). These results showed that either glutaminase or glutamate dehydrogenase inhibition caused an enhancement of NRF2 protein levels. In contrast, the effect of the inhibition of glutamine transporter ASCT2 on NRF2 protein levels is cell-dependent since other glutamine transporters can be active. Therefore, the effect of glutamine deprivation can be mimicked with specific inhibitors that can be more applicable in terms of cancer therapy. To sum up, we found that glutamine deprivation in HT29 and HCT116 cells elevates ROS levels, increasing oxidative stress. Augmented oxidative stress inactivates KEAP1 leading to the release and accumulation of NRF2 transcription factor, which triggers the increase of *G6PD* expression to balance the enhanced oxidative stress.

## 4. Discussion

Several tumors have a higher dependence on the PPP, particularly on the oxidative phase of the PPP, compared to non-transformed cells [8,11,12,43]. G6PD upregulation has been proposed as an indicator of poor prognosis in several types of cancers, including colon cancer [7,43,44,45]. Epidemiological data have also shown that G6PD deficiency is associated with a reduction in colorectal cancer risk [46] and in susceptibility to cancer of endodermal origin [47]. Moreover, in the last years, there is increasing evidence proving that G6PD is a major contributor to invasion, migration, and metastasis in several cancers [45,48,49,50]. Therefore, G6PD has been proposed as an attractive therapeutic target in the fight against cancer in several studies [44,45,51] as it plays an important role in the biosynthesis of ribose and the production of NADPH, which is necessary for the regulation of ROS levels [45,52]. However, the entire mechanism by which G6PD inhibition affects cancer progression is not fully elucidated, and until the present, there are no conclusive studies demonstrating why G6PD is essential for cancer cells. In fact, we have observed that reducing the activity of G6PD by over 75% only decreased cells’ proliferation by over 20%, and it has been hypothesized that the demand for products of the G6PD reaction can be fulfilled by compensatory mechanisms such as the malic enzyme, isocitrate dehydrogenase or folate metabolism for NADPH production, by transketolase for ribose-5-phosphate synthesis, and by nutrient scavenging from the microenvironment. On the other hand, G6PD is likely to be particularly important in the context of specific tumor types or genetic events such as NRF2 activation [48]. This evidence has driven in the last years an important ongoing area of investigation devoted to identifying tumors that are particularly sensitive to G6PD inhibition, unveiling an urgent need to explore the impact of tumor microenvironment nutrients on G6PD upregulation in cancer.

Glutamine, on the other hand, is a versatile nutrient having vital importance for most cancer cells. In fact, there are both in vivo and in vitro studies previously conducted that demonstrate the importance of glutamine for cancer cells [53]. In this regard, the enzyme glutaminase (GLS1), which catalyzes the first step of glutamine metabolism, is highly expressed in colon cancer and linked to significantly reduced survival [54,55]. Besides, recent epidemiological studies correlate low serum glutamine levels (indicative of higher glutamine consumption) with poorer overall survival in colorectal cancer patients [56]. Even though many studies have been conducted to unveil the metabolism of glutamine in tumors, there is much yet to be explored. Additionally, several clinical studies with GLS1 inhibitors (Phase I/II clinical trial: telaglenastat, CB-839) [55] for the treatment of different types of cancer, including colorectal cancer, showed promising results. Moreover, recent studies suggest targeting glutamine mitochondrial transporters as a new cancer starvation strategy for controlling tumor growth [57].

Here, we first showed that G6PD has an important role in the proliferation of HT29 and HCT116 colon cancer cells. *G6PD* gene expression was reduced by more than 90% by employing RNA interference technology, which, in turn, reduced the activity of this enzyme by about 80%. This reduction led both cell lines to a decrease in proliferation by about 25%, highlighting the importance of this enzyme in the proliferation of colon cancer cells. Considering that G6PD usually only operates around 2% of its maximum potential in non-transformed cells [58] while in cancer cells this percentage is significantly higher, it is vital to highlight the key role of G6PD in the proliferation of tumor cells. Indeed, several other studies in various tissues also demonstrated the importance of G6PD in cell viability [10,45,59].

Concerning glutamine depletion, we observed that glutamine withdrawal significantly reduced the proliferation of colon cancer cells at various time points. Li et al. have demonstrated that colon cancer cells, increase their glutamine metabolism when glucose is scarce in the microenvironment to support survival [60]. In contrast, cells cultured without glutamine do not use other nutrient sources of the culture medium to maintain the proliferation rate since they enter a quiescent state without exhibiting cell death, as reported by the increase of cells in the G1 phase.

We also measured the population of the cells in each phase of the cell cycle, and we observed that both HT29 and HCT116 cells with reduced G6PD activity were arrested in the S and G2 phases of the cell cycle. In fact, since cells must have enough nucleotides to overcome the checkpoint at the G1 phase [8], the decreased rate of precursors for nucleotides synthesized in the PPP must have been compensated by the activation of the non-oxidative pathway of the PPP. Besides that, Saqcena et al. reported that DNA damaging agents selectively induce apoptosis in cancer cells arrested in the S and G2 phases of the cell cycle, implying that phase-specific cytotoxic drugs in combination with G6PD inhibitors may create synthetic lethality that can be a promising therapeutic approach in the combat against cancer [61]. On the other hand, we observed that glutamine withdrawal arrested both colon cancer cell lines in the G1 phase, which is in concordance with the described key roles of glutamine in the transition from G1 to S phase in cell cycle and the nucleotide synthesis and also with the reduction in proliferation without increasing cell death [62].

Even though the oxidative phase of the PPP is the primary source of NADPH required for redox detoxification and several other key biosynthetic processes, the cytosolic isoform of the malic enzyme (ME1) and NADP-dependent isocitrate dehydrogenase (IDH) are additional sources of NADPH in the cells, also playing a major role in glutamine metabolism [9,63]. Associated with this, Jiang et al. have demonstrated that the inhibition of both the oxidative phase of the PPP and IDH enhances ROS levels in lung cancer cells [64]. Similarly, we have previously reported an increased glutamine consumption in breast cancer cells with ablated 6-phosphogluconate dehydrogenase, the third enzyme of the oxidative PPP [9], and that glutamine deprivation enhances ME flux in breast cancer cells [65], indicating strong crosstalk between glutamine metabolism and ox-PPP. Thus, we hypothesized that there might be a relation between G6PD and glutamine-dependent reactions for NADPH production.

To this end, we deprived cells of glutamine and observed that glutamine withdrawal led to an increase in both the expression and the enzyme activity of G6PD at different time points. Hence, we evidenced a strong link between G6PD and glutamine availability. In addition, Son et al. previously reported that besides feeding mitochondrial reactions, glutamine is also a substrate for the malic enzyme, which converts malate to pyruvate for NADPH production, and that withdrawal of glutamine from the cell environment increases ROS levels [66]. Accordingly, in our case, the deprivation of glutamine in HT29 and HCT116 cells increased the expression and enzyme activity of G6PD as well as the intracellular ROS levels.

It has been extensively described that the NRF2 transcription factor plays a key role in regulating ROS and NADPH balance [67], controlling G6PD expression and activity as well [23]. Reasonably, we hypothesized that NRF2 might mediate the reported enhancement of G6PD. Our results confirm that glutamine depletion induced the overexpression of NRF2 protein, as well as a significant increase of gene expression levels of *HMOX1* and *NQO1*, which are NRF2 target genes. Thus, we confirm the relation between glutamine availability and G6PD activity mediated by the NRF2 transcription factor.

Taking into account that glutaminase inhibition monotherapy is known to be insufficient and that GLS inhibitors are increasingly used in combination with other cancer therapies [68], our results suggest that a combination of GLS and G6PD inhibitors could be a promising strategy to target glutamine addiction in colon cancer and to disrupt ROS balance efficiently. This aligns with a recent report showing that the effect of the glutaminase inhibitor CB-839 on liver cancer can be further enhanced by several anti-metabolic drugs such as oxidative phosphorylation (OXPHOS) inhibitor IACS-10759, and G6PD inhibitor Dehydroepiandrosterone (DHEA) [69], which reinforces the idea that the unveiled dependence of G6PD expression on glutamine deprivation mediated by NRF2 pathway can open new avenues in the design of combined treatments to target glutamine addiction in colorectal cancer. Considering that nowadays Positron emission tomography (PET)-based methods monitoring glutamine and glucose are available [70,71], it is envisioned that in the near future, the use of these methods may facilitate the translation of the findings described here to the identification of those tumors that are most likely to benefit from combined therapies targeting G6PD and glutamine addiction.

## 5. Conclusions

In this study, we manifestly showed the importance of the PPP enzyme G6PD and glutamine for the proliferation and survival of colon cancer cells. We demonstrated that both G6PD inhibition and glutamine deprivation led colon cancer cells into cell cycle arrest and subsequent decrease in proliferation. Also, we unveiled a novel relationship between glutamine availability and G6PD, the gate-keeping enzyme of the PPP oxidative phase. We showed that G6PD is overexpressed in colon cancer cells upon glutamine withdrawal following an increase in ROS and NRF2 protein levels. Finally, we propose the potential of inhibiting together the oxidative PPP and glutamine catabolism as a therapeutic strategy in colorectal glutamine-addicted tumors.

## Data Availability

Data is contained within the article and supplementary material.

## Supplementary Materials

The following material is available online: https://www.mdpi.com/article/10.3390/antiox10091349/s1. Figure S1. Intensity ratio of western blots of Figure 1 and Figure 2. Figure S2. Cell Cycle Histograms. Figure S3. Intensity ratio of western blots of Figure 5.
