# Supplementary material for: Glutamine Modulates Expression and Function of Glucose 6-Phosphate Dehydrogenase via NRF2 in Colon Cancer Cells
Source: Antioxidants (Basel). 2021 Aug 25;10(9):1349. doi: 10.3390/antiox10091349 (PMC8472416; doi:10.3390/antiox10091349)
Supplement: Supplementary file 1 [file antioxidants-10-01349-s001.zip › antioxidants-1351655-supplementary.pdf]

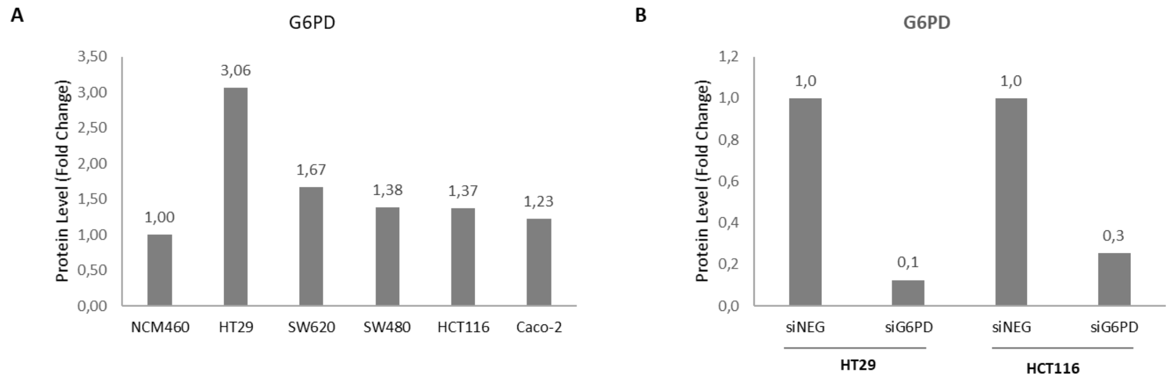

Supplementary Figure S1. Intensity ratio of western blots

Intensity ratio of each band on the western blot was calculated using ImageJ® Software (public domain National Institutes of Health, USA, http://rsbweb.nih.gov/ij/) and normalized to its corresponding β-actin. A: Intensity ratios of G6PD in 6 colon cancer cell lines. Fold change is calculated considering the NCM460 cell line as control. B: Intensity ratios of G6PD in HT29 and HCT116 cells after treatment of siNEG or siRNA targeting 6PGD and relative to siNEG condition.

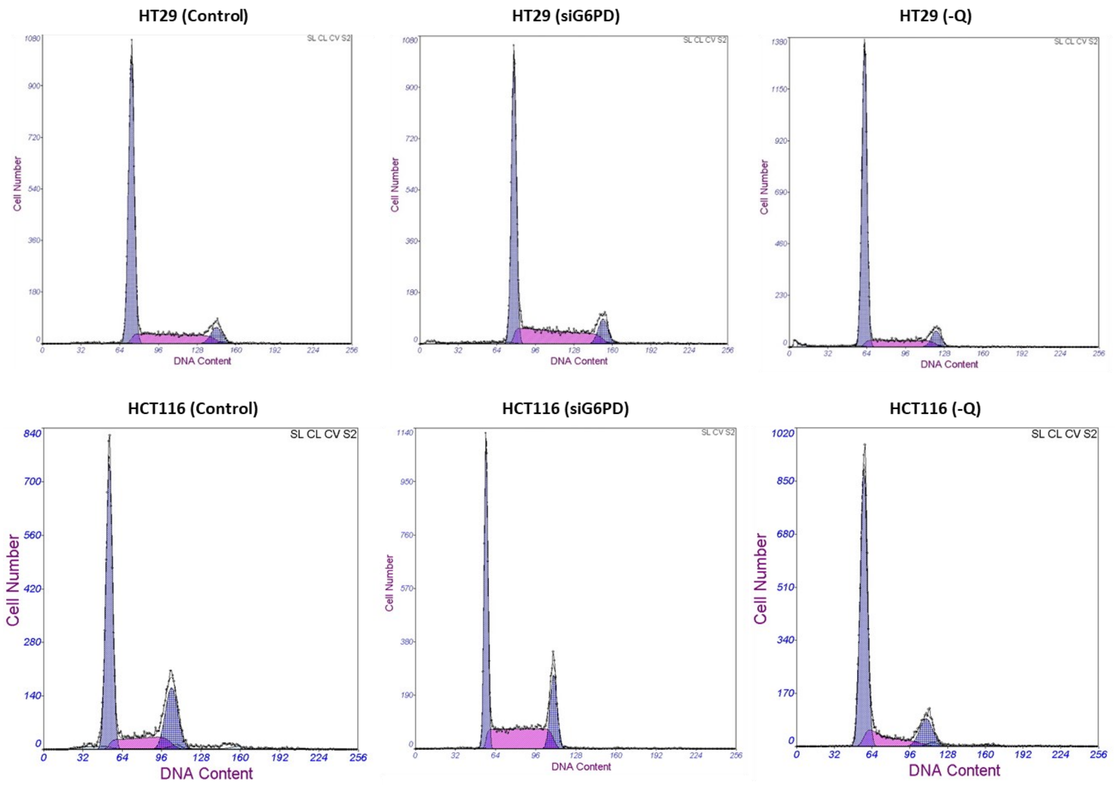

Supplementary Figure S2. Cell Cycle Histograms

Cell cycle analysis was performed using a Beckman Coulter® Epics® XLTM Flow Cytometer with a cut-off at 1×10^4 cells. The cell cycle distribution analysis was done using FlowJo® software to obtain the percentage of cells in G1, S, and G2 phases. One example of each condition is shown.

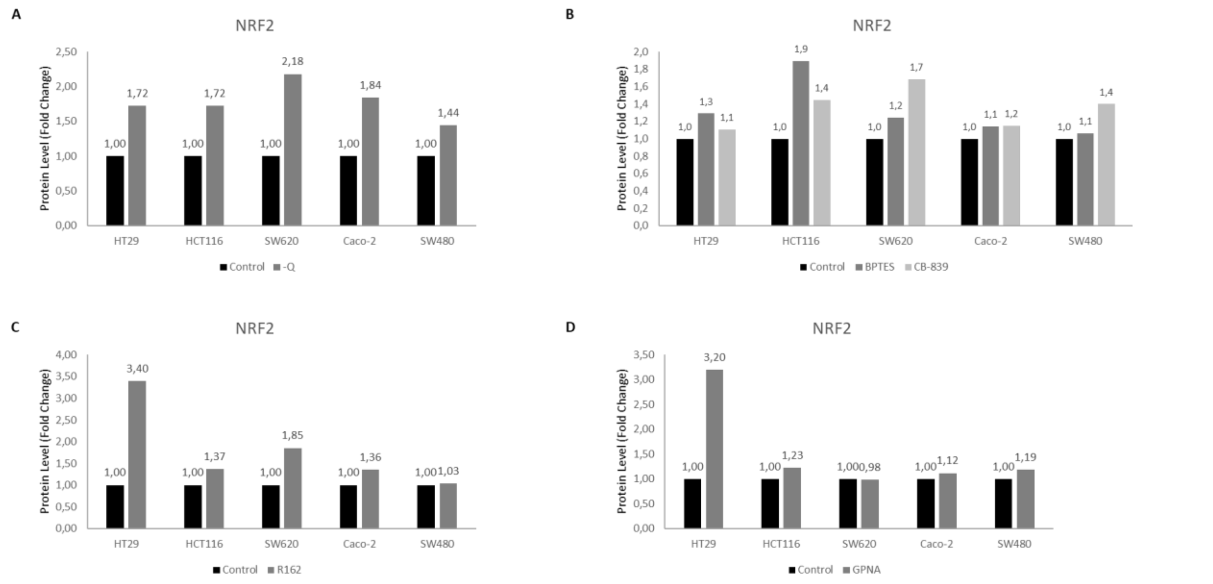

Supplementary Figure S3. Intensity ratio of western blots

Intensity ratio of each band on the western blot was calculated using ImageJ® Software (public domain National Institutes of Health, USA, http://rsbweb.nih.gov/ij/) and normalized to its corresponding β-actin. Intensity ratios of NRF2 in 5 colon cancer cell lines A: upon glutamine deprivation; B: upon glutaminase inhibition; C; upon glutamate dehydrogenase inhibition and D: upon glutamine transporter inhibition. Fold changes were calculated considering non-treated cells as control.
